# Intramuscular methylergonovine to decrease blood loss during cesarean delivery for twins: A triple‐blinded placebo‐controlled randomized trial

**DOI:** 10.1002/pmf2.12039

**Published:** 2025-01-27

**Authors:** Helen B. Gomez Slagle, Russell Miller, Shai Bejerano, Dena Goffman, Mary E. D'alton, Mirella Mourad

**Affiliations:** ^1^ Division of Maternal‐Fetal Medicine, Department of Obstetrics and Gynecology NewYork‐Presbyterian Hospital, Columbia University Irving Medical Center New York New York USA; ^2^ Department of Obstetrics and Gynecology Vagelos College of Physician and Surgeons, Columbia University Irving Medical Center New York New York USA

**Keywords:** cesarean delivery, hemoglobin, methylergonovine, twins

## Abstract

**Objective:**

Our objective was to evaluate whether prophylactic administration of intramuscular (IM) methylergonovine after cord clamping reduces blood loss during planned cesarean delivery (CD) of twin pregnancies.

**Method:**

This single‐center randomized placebo‐controlled triple‐blinded trial compared the effects of IM methylergonovine versus saline placebo control on maternal blood loss at CD. Pregnant individuals with twin gestations at ≥ 34 weeks of gestation undergoing planned CD were enrolled. Methylergonovine or saline placebo was administered immediately after umbilical cord clamping of the second twin in addition to standard oxytocin prophylaxis. The primary outcome was a change in mean maternal hemoglobin (Hgb) level from preoperative to postoperative day 1 (POD 1). Secondary outcomes include intraoperative blood loss, additional uterotonic use, and rates of postpartum hemorrhage and transfusion. We planned to enroll 66 patients to detect a standard deviation (SD) greater drop in postoperative maternal Hgb, assuming −1.40 (± 0.83) g/dL Hgb difference after planned CDs (90% power, alpha 0.05). Pearson chi‐square test, Fisher's exact test, *t*‐test, and Wilcoxon rank sum test with intent‐to‐treat principles were performed as appropriate. A linear regression model was performed on the primary outcome.

**Results:**

A total of 66 patients (33 methylergonovine and 33 placebo) were randomized. There were no demographic differences between methylergonovine and placebo groups. Median gestational age at delivery was 37 weeks for both groups. Mean Hgb drop on POD 1 was 1.1 g/dL (SD 0.7 g/dL) for methylergonovine and 2.1 g/dL (SD 0.9 g/dL) for placebo (*p* < 0.001). Quantitative blood loss was significantly lower in patients receiving prophylactic methylergonovine (891 vs. 1017 mL, *p* = 0.003). Postpartum hemorrhage rates were lower in the methylergonovine group (18.2% vs. 54.5%, *p* = 0.002). There were seven cases of unblinding due to hemorrhage in the placebo group compared to no cases in the methylergonovine group (*p* = 0.01); all unblinded subjects received methylergonovine.

**Conclusion:**

Prophylactic IM methylergonovine administered after umbilical cord clamping during twin CD significantly reduced intraoperative blood loss and hemoglobin drop on POD 1. These data support further study of methylergonovine as a preventative treatment strategy at the time of twin CD.

## 1 INTRODUCTION

Twin pregnancy poses unique challenges to obstetrical management. Specifically, uterine overdistension can impair myometrial contractility following delivery, resulting in higher rates of atony and postpartum hemorrhage (PPH) [1]. Individuals with twin pregnancy undergoing cesarean delivery (CD) experience higher rates of postpartum hemorrhage compared to singleton pregnancy [2]. Despite advancements in the obstetric management of PPH, hemorrhage after delivery remains a significant contributor to maternal morbidity and mortality [3, 4]. Therapeutic adjuncts to oxytocin are needed when uterine atony is refractory to initial therapy. Methylergonovine is a fast‐acting, semisynthetic ergot alkaloid that has been shown to minimize intraoperative blood loss when used prophylactically in singleton pregnancies [5]. Therefore, the objective of this study is to evaluate whether prophylactic administration of intramuscular methylergonovine after cord clamping reduces blood loss during CD in twin pregnancies.

## 2 MATERIALS AND METHODS

This randomized placebo‐controlled triple‐blinded trial was conducted from February 2023 through March 2024 at a single tertiary care teaching hospital in New York. Prior to the initiation of the study, approval was obtained from the local Institutional Review Board (AAAU3902) and the trial was registered with ClinicalTrials.gov (NCT05772156). This study was a randomized controlled trial for individuals pregnant with twin gestations undergoing scheduled cesarean.

### 2.1 Participants

Pregnant individuals ≥ 18 years of age with a twin gestation at or beyond 34 weeks of gestation were eligible for the trial. Patients undergoing a planned CD were included, regardless of chorionicity. Patients were screened electronically by clinical research staff for eligibility. Eligible patients were approached and consented to participate in the study shortly after their arrival at the perioperative anesthesia care unit for scheduled CD.

Individuals with placental or uterine anomalies, including placenta accreta spectrum, were excluded from study participation. Additionally, patients with contraindications to methylergonovine exposure including a history of chronic hypertension, hypertensive disorders in pregnancy, coronary artery disease, protease inhibitor use, or known hypersensitivity to methylergonovine were excluded. Hypertension was defined as pre‐existing chronic hypertension when diagnosed before 20 weeks of gestation or gestational hypertension when diagnosed after 20 weeks of gestation using standard blood pressure and laboratory criteria [6].

### 2.2 Randomization

Participants were sequentially assigned to the methylergonovine or placebo arms using computer‐generated random groups with a 1:1 allocation ratio [7], prepared by a data analyst with no clinical involvement in the trial. The randomization was conducted in R, using the randomizr package version 0.22.0 [8]. A clinician with no involvement in a study participant's care prepared the study solution using the assignment.

### 2.3 Study procedures

As is standard institutional practice for all scheduled CDs, preoperative hemoglobin level was assessed on a complete blood count obtained within 72 h prior to delivery. CDs were performed by residents, fellows, and attending physicians, with appropriate supervision. All study participants received neuraxial anesthesia for surgery. Following standard institutional protocol, a 500‐mL bag containing 30 units of oxytocin was infused at 500 mL/h for 1 h, followed by 125 mL/h (7.5 units) for the following 4 h after cord clamping of the second twin. Adjustments to the standard oxytocin administration were made at the discretion of the providers. The oxytocin infusion was continued throughout the procedure and during the patient's postoperative recovery stay for a total of 4 h after delivery per standard protocol.

Immediately after cord clamping of the second twin, patients received either intramuscular methylergonovine 0.2 mg (1 mL) or intramuscular placebo (normal saline, 1 mL). The methylergonovine and placebo solutions were visually indistinguishable clear solutions contained in a labeled syringe that listed “Study Drug,” participant ID, and preparation date and time, but not the syringe contents. The study medication and placebo were delivered intramuscularly into the vastus lateralis muscle after umbilical cord clamping of the second twin. Delayed cord clamping was not performed. All clinicians, research personnel collecting delivery data, and patients were blinded to treatment allocation.

Additional uterotonics or tranexamic acid (TXA) were administered at the discretion of the primary provider using standard dosing and frequency recommended by the American College of Obstetricians and Gynecologists (ACOG) [9]. An increase in the oxytocin infusion rate was not considered an additional uterotonic agent, and this was also utilized at the direction of the primary provider. Unblinding was performed immediately upon the request of the providers at their discretion.

All participants received routine postoperative care. This included administration of intravenous fluids (125 mL/h lactated ringers) until tolerating oral fluids. On postoperative day 1, maternal hemoglobin level was assessed on a complete blood count.

### 2.4 Outcome assessment

The primary outcome was an objective measure of maternal blood loss: the change in maternal hemoglobin level on postoperative day 1 compared with preoperative hemoglobin level. Quantitative blood loss was determined by measuring suction cannister contents subtracting amniotic fluid and irrigation volume and weighing soiled surgical sponges, drapes, and pads as is standard institutional practice for all CDs [10, 11, 12]. Additional secondary outcome hemorrhage metrics included estimated blood loss, postpartum hemorrhage (defined as quantitative blood loss > 1000 mL), additional uterotonic administration, and the need for blood transfusion. Additional outcomes were collected including surgical time, use of intrauterine balloon tamponade or vacuum‐induced uterine tamponade device placement, B‐lynch suture placement, and the use of hemostatic sealant agents.

### 2.5 Statistical analysis and sample size calculations

Based on a prior study of elective CDs in twins, a mean change and standard deviation (SD) in hemoglobin level of −1.37 (0.87) g/dL was estimated [13]. A 20% crossover rate from the placebo group to the methylergonovine group was anticipated. The investigators selected a 1‐SD greater drop in postoperative maternal hemoglobin level with placebo as meaningful for three reasons: first, this definition has been previously used in published reports [14]; second, there is no accepted definition of a minimum clinically important difference in postoperative hemoglobin change; and third, given the burden of maternal morbidity and mortality due to postpartum hemorrhage, a 1‐SD difference might alter clinical decision‐making. Assuming 90% power, equal group sizes, and a two‐sided *p*‐value, we assumed 33 patients in each group for a total sample size of 66 subjects.

The data from all participants were analyzed according to the group to which they were randomly assigned. Primary outcome data were available for all participants. Categorical variables were analyzed using the chi‐square test or Fisher's exact test as appropriate. Continuous variables were assessed using either the *t*‐test or Wilcoxon rank sum test where appropriate. In addition, a linear regression model was fit for the primary outcome of difference in hemoglobin levels between preoperative and postoperative day 1. The model was adjusted for maternal age at delivery, pregravid body mass index, prior number of cesareans, and anterior placenta. All hypothesis tests were two‐sided and significance was set at *p* < 0.05. An internal data and safety monitor (Quality Assurance Monitor for the Department of Obstetrics and Gynecology) performed monitoring. There were no interim analyses. Statistical analyses were performed using RStudio version 2024.04.2 Build 764 (Posit Software, PBC, Boston, MA, USA) and R version 4.4.1. (R Foundation for Statistical Computing, Vienna, Austria).

## 3 RESULTS

### 3.1 Participant characteristics

**FIGURE 1:**
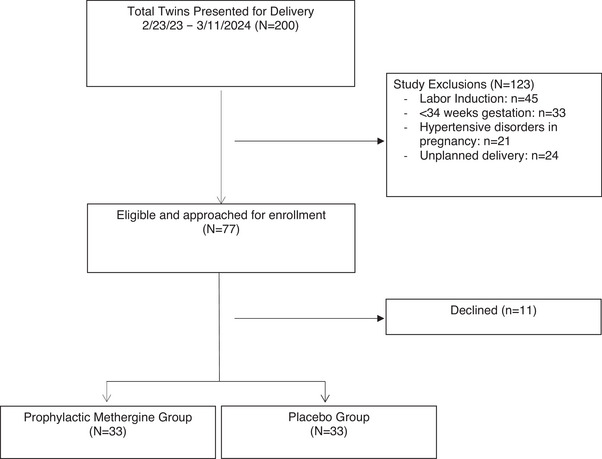
CONSORT participant flow chart.

Between February 2023 and March 2024, 200 patients delivered twins, 123 of which were not eligible for recruitment (Figure 1). Common reasons for exclusion included: labor induction, < 34‐week delivery, and hypertensive disorders of pregnancy. Of the 77 individuals who met eligibility criteria and were approached for participation, 11 patients declined involvement. A total of 66 patients were consented and randomized into the two treatment groups.

Patient demographic characteristics by treatment group are provided in Table 1. There were no demographic differences between groups. The median gestational age at trial entry, and thus delivery, was 37.0 weeks in both groups. There were no cases of prior postpartum hemorrhage among study participants. The mean (SD) preoperative hemoglobin level was 11.6 (1.2) g/dL in the methylergonovine group and 12.0 (1.3) g/dL in the placebo group.

**TABLE 1:** Characteristics by randomized treatment group.

	Methylergonovine (*n* = 33)	Placebo (*n* = 33)
Age (years), mean, SD	33.4 (6.2)	32.8 (6.7)
Gestational age at induction (weeks), median, IQR	37.0 (35.7 to 38.0)	37.0 (35.4 to 37.6)
Nulliparous, *n* (%)	15 (45.5)	15 (45.5)
Race		
White	22 (66.7)	22 (66.7)
Black	11 (33.3)	9 (27.3)
Asian	0 (0.0)	2 (6.1)
Hispanic ethnicity, *n* (%)	15 (45.5)	14 (42.4)
Number of prior cesarean deliveries, *n* (%)		
0	23 (69.7)	24 (72.7)
1	7 (21.2)	7 (21.2)
≥2	3 (9.1)	2 (6.1)
Chorionicity, *n* (%)		
Dichorionic	21 (63.6)	21 (63.6)
Monochorionic–diamniotic	12 (36.4)	12 (36.4)
BMI (kg/m^2^), mean, SD	30.0 (5.7)	30.5 (7.3)
Tobacco use in pregnancy, *n* (%)	1 (3.0)	0 (0.0)
Gestational diabetes mellitus, *n* (%)	5 (15.2)	6 (18.2)
Preoperative maternal hemoglobin level (g/dL), mean (SD)	11.6 (1.2)	12.0 (1.3)

Abbreviations: IQR, interquartile range; SD, standard deviation.

Surgical characteristics for each group are shown in Table 2. All cases were performed under neuraxial anesthesia. Delivery was via a low transverse hysterotomy in all but 1 case in the placebo group in which a J incision was required. Surgical sterilization was performed in five participants in the methylergonovine group and four participants in the placebo group.

**TABLE 2:** Surgical characteristics by randomized treatment group.

	Methylergonovine (*n* = 33)	Placebo (*n* = 33)	*p*
Indication for cesarean delivery			1.0
Elective	9 (27.3)	10 (30.3)	
Malpresentation of presenting twin	8 (24.2)	7 (21.2)	
Repeat	10 (30.3)	9 (27.3)	
Abnormal fetal testing	4 (12.1)	5 (15.2)	
Vasa/placenta previa	0 (0.0)	1 (3.0)	
History of myomectomy	2 (6.1)	1 (3.0)	
Spinal anesthesia	33 (100.0)	33 (100.0)	–
Hysterotomy type			1.0
Low transverse	33 (100.0)	32 (97.0)	
Classical	0 (0.0)	0 (0.0)	
T‐ or J‐incision	0 (0.0)	1 (3.0)	
Anterior placenta	22 (66.7)	21 (63.6)	0.80
Surgical sterilization	5 (15.2)	4 (12.1)	1.0

### 3.2 Primary outcome

**FIGURE 2:**
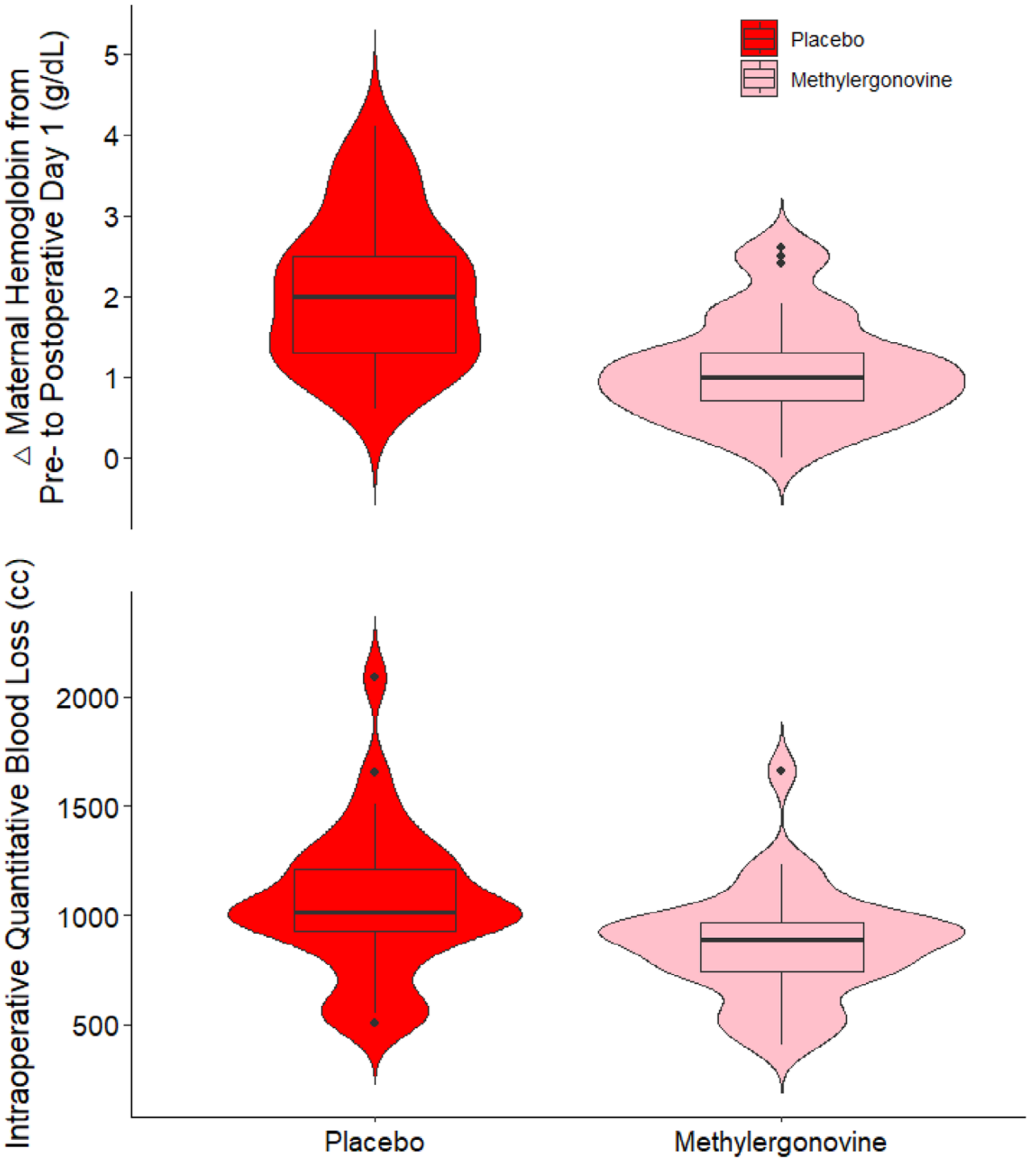
Violin plots with superimposed box plots of change in hemoglobin and intraoperative blood loss by randomized treatment group. Violin plots display the density of the overall data, and boxplot boxes display the median and interquartile range.

The change in maternal hemoglobin level from preoperative to postoperative day 1 was different between groups, with a mean decrease of −1.1 g/dL (−0.7 g/dL) in the methylergonovine group and −2.1 g/dL (−0.9 g/dL) in the placebo group (*p* < 0.001) (Table 3). When adjusting for potential confounders in the linear regression model, there was a 1.0 g/dL decrease on average (95% CI: −1.40, −0.59; *p* < 0.001) in the change of maternal hemoglobin from preoperative to postoperative day 1 for methylergonovine compared to placebo. A violin plot with superimposed box plots of the primary outcome is shown in Figure 2; violin plots display the density of the overall data, and boxplot boxes display the median and interquartile range.

**TABLE 3:** Surgical outcomes by randomized treatment group.

	Methylergonovine (*n* = 33)	Placebo (*n* = 33)	*p*
Change in hemoglobin (g/dL), mean ± SD	1.1 ± 0.7	2.1 ± 0.9	<0.001
Total surgical time (minutes), median [IQR]	62.0 (54; 75)	61.0 (49; 69)	0.79
Estimated blood loss (mL), median [IQR]	800.0 [800.0; 900.0]	1000.0 [800.0; 1098.0]	0.003
Quantitative blood loss (mL), median [IQR]	891.0 [743.0; 966.0]	1017.0 [931.0; 1211.0]	0.003
Postpartum hemorrhage^a^	6 (18.2)	18 (54.5)	0.002
Intraoperative prostaglandin administration	0 (0.0)	2 (6.1)	0.49
Intraoperative carboprost tromethamine administration	1 (3.0)	4 (12.1)	0.36
Intraoperative tranexamic acid administration	2 (6.1)	7 (21.2)	0.15
Intraoperative use of two or more medications (uterotonic agents or antifibrinolytic)	0 (0.0)	6 (18.2)	0.02
Unblinding and intraoperative methylergonovine administration	0 (0.0)	7 (21.2)	0.01
Intraoperative hemostatic floseal	1 (3.0)	2 (6.1)	1.0
B‐Lynch placement	0 (0.0)	1 (3.0)	1.0
Composite number of surgical or radiological interventions (laparotomy, evacuation of hematoma, uterine packing, intrauterine balloon tamponade, interventional radiology)	0 (0.0	0 (0.0)	–
Intraoperative intravenous fluid volume (cc), mean ± SD	1708.7 ± 747.2	1871.9 ± 752.5	0.38
Postpartum infection complications^b^	0 (0.0)	2 (6.1)	0.49
Postpartum gestational hypertension	3 (9.1)	2 (6.1)	1.0
Postpartum preeclampsia	3 (9.1)	4 (12.1)	1.0
Length of stay (days), median [IQR]	4.0 [3.0; 4.0]	4.0 [4.0; 6.0]	<0.001
Hospital readmission	1 (3.0)	3 (9.1)	0.61
Blood transfusion	2 (6.1)	4 (12.1)	0.67
Transfusion‐related acute lung injury	0 (0.0	0 (0.0)	–
Acute elevation of serum creatinine of ≥ 0.3 mg/dL	0 (0.0	0 (0.0)	–
Admission to the intensive care unit for more than 24 h	0 (0.0	0 (0.0)	–
Hysterectomy	0 (0.0)	0 (0.0)	–

*Note*: Data are presented as *N* (%), unless otherwise indicated.

Abbreviations: IQR, interquartile range; SD, standard deviation.

^a^ Defined as quantitative blood loss of greater or equal to 1000 mL.

^b^ Defined as endometritis, surgical site infection, or pelvic abscess.

### 3.3 Secondary outcomes

The surgical time was not statistically significantly different between groups (*p* = 0.79), with a median of 62.0 min (IQR, 54.0, 75.0) in the methylergonovine group and 61.0 min (IQR, 49.0, 69.0) in the placebo group. One B‐Lynch suture was performed in a patient receiving placebo. Intraoperative use of two or more medications, including uterotonic or antifibrinolytic agents, occurred in no patients in the methylergonovine and six patients who received placebo (0% vs. 18%, *p* = 0.02, Table 2). Quantitative blood loss was significantly lower in the methylergonovine group with a median quantitative blood loss of 891 mL [IQR 743, 966] compared to 1017 mL [931–1211] in patients allocated to placebo (*p* = 0.003) (Table 3). Participants receiving methylergonovine had lower estimated blood loss (800 vs. 1000 mL, *p* = 0.003). There was a lower incidence of postpartum hemorrhage (6% vs. 18%, *p* = 0.002) in the participants receiving methylergonovine. Four participants in the placebo group and two participants in the methylergonovine required transfusion (*p* = 0.67). There were seven cases of unblinding and methylergonovine use due to hemorrhage in the placebo group compared to no cases of unblinding in the methylergonovine group (*p* = 0.01) (Table 4).

**TABLE 4:** Neonatal outcomes by randomized treatment group.

	Methylergonovine (*n* = 66)	Placebo (*n* = 66)	*p*
Combined infant weight, Mean, SD	4694 (521.0)	4684 (813.4)	0.95
Neonatal weight, mean, SD	2347 (329.1)	2342 (445.0)	0.94
APGAR scores 1 min, Median, IQR	8.0 (8.0 to 9.0)	8.0 (8.0 to 8.0)	0.38
APGAR scores 5 min, Median, IQR	9.0 (9.0 to 9.0)	9.0 (9.0 to 9.0)	0.80
NICU Admission	19 (28.8)	20 (30.3)	0.85

*Note*: This considers each twin as an observation in the database, *n* = 132. Correlations between twins with the same birthing person were not accounted for in the analysis.

Abbreviations: IQR, interquartile range; SD, standard deviation.

There were no cases of postpartum infection, hysterectomy, intrauterine balloon or vacuum tamponade, interventional radiology, or intensive care unit admission in the study groups. Length of stay and readmission rates were not significantly different between groups. There was no significant difference between study groups in the incidence of postpartum gestational hypertension or postpartum preeclampsia.

## 4 DISCUSSION

### 4.1 Principle findings

Prophylactic intramuscular methylergonovine administered after umbilical cord clamping during twin CD significantly reduced intraoperative blood loss, rates of postpartum hemorrhage, and hemoglobin drop at postoperative day 1.

### 4.2 Results in the context of what is known

Comparisons with the existing literature are challenging because twin pregnancies have largely been excluded from trials assessing the prophylactic effect of uterotonic medications [5, 17] and no subgroup analyses have assessed the utility of these medications in twins [18, 19]. In a secondary analysis of the TRAAP trial [20], Sentilhes et al. found no difference in blood transfusion or hemoglobin drop in patients receiving tranexamic acid compared to placebo in twin gestations [21].

Individuals with twin pregnancies are a particularly vulnerable group, as their risk of severe postpartum hemorrhage is four to five times higher than that of singleton pregnancies [15] and they give birth more often by CD, yet no specific prophylaxis is recommended for this population [16].

Methylergonovine is thought to elicit its uterotonic effects through stimulation of the a‐adrenergic and serotonin receptors in the myometrium [22]. Masse et al. found that prophylactic methylergonovine following unplanned CD for singleton gestations is associated with reduced need for additional uterotonic agents, lower mean quantitative blood loss, and a lower frequency of blood transfusion [5]. While multiple gestations were excluded from this study, we have demonstrated here that methylergonovine appears to be a comparably promising and effective prophylactic measure in twin gestations.

Methylergonovine was selected as the uterotonic agent for this study due to its rapid onset of action and established efficacy in preventing and managing postpartum hemorrhage, particularly in cases of uterine atony. Twin pregnancies, which are associated with increased uterine distention and a higher risk of hemorrhage, may particularly benefit from a fast‐acting agent like methylergonovine. While misoprostol is also an effective uterotonic agent and generally associated with a lower cost and fewer side effects, its slower onset of action makes it less suitable for managing surgical bleeding. Furthermore, compared to 15‐methyl prostaglandinF2a, methylergonovine is more cost‐effective and easier to manage in clinical settings. 15‐Methyl prostaglandinF2a requires cold storage, which can present logistical challenges, especially in emergency situations, and is associated with a higher incidence of gastrointestinal side effects which further influenced our choice of methylergonovine.

Although methylergonovine should be avoided in patients with pregnancies complicated by maternal hypertensive disorders or other rare contraindications, for most pregnant patients our results suggest that prophylactic methylergonovine can be given without concern for a clinically significant rise in blood pressure.

### 4.3 Clinical implications

PPH is the leading global cause of maternal morbidity and mortality [3, 4] and there is a critical need for effective adjuncts to oxytocin for the prevention of bleeding. We found that prophylactic methylergonovine may be helpful in twin gestations undergoing a planned cesarean.

### 4.4 Research implications

Larger‐randomized controlled trial studies are required to confirm whether methergine or other prophylactic uterotonic medication administration results in a lower incidence of postpartum hemorrhage after vaginal delivery and unplanned CD. Additionally, further research is needed to determine whether prophylactic methylergonovine administration for the prevention of blood loss in twins is cost‐effective.

### 4.5 Strengths and limitations

A major strength of this study lies in its triple‐blind, randomized controlled trial design. Providers, patients, and data analysts were blinded to treatment allocation which reduced bias as compared to prior trials. Additionally, we used an objective measure of blood loss as the primary outcome. Furthermore, our study occurred in a generalizable clinical setting with diverse patient populations.

Our study has several limitations. First, we focused on the prophylactic use of methylergonovine and did not assess the effects of other uterotonic agents such as 15‐methyl prostaglandinF2a. While no rigorous data support the use of any one uterotonic agent as an additional drug to treat uterine atony, and current choice among the available agents is typically dictated by physician preference and patient contraindications to specific medications, methylergonovine may be challenging in twins given higher rates of hypertensive disorders in pregnancy within this population [23]. We excluded 21 patients (10.5%) from participation due to hypertensive disorders in pregnancy. Other limitations of this study include its relatively small size and only including CDs in our cohort.

## 5 CONCLUSION

We found that prophylactic methylergonovine administered after umbilical cord clamping during twin CD significantly reduced intraoperative blood loss and postdelivery hemoglobin drop. These data support further study of methylergonovine as a preventative treatment strategy at the time of twin CD with the potential to reduce the risk of postpartum hemorrhage and associated sequelae in this at‐risk patient population.
